# Topic Modeling Analysis of Diabetes-Related Health Information during the Coronavirus Disease Pandemic

**DOI:** 10.3390/healthcare11131871

**Published:** 2023-06-27

**Authors:** Soyoon Min, Jeongwon Han

**Affiliations:** College of Nursing Science, Kyung Hee University, 26, Kyunghee-daero, Dongdaemun-gu, Seoul 02447, Republic of Korea; papaugini@naver.com

**Keywords:** coronavirus disease, diabetes, health information, topic modeling

## Abstract

This study aimed to provide diabetes-related health information by analyzing queries posted in the diabetes-related online community required during the COVID-19 pandemic. A total of 9156 queries from the diabetes-related online community, dated between 1 December 2019 and 3 May 2022, were used in the study. The collected data were preprocessed for bidirectional encoder representation from transformer topic modeling analysis. Topics were extracted using the class-based term frequency–inverse document frequency for nouns and verbs. From the extracted verbs, words with common definitions were subject to substitution and unification processes, which enabled the identification of multifrequent verb categories by noun topics. The following nine noun topics were extracted, in this order: dietary management, drug management, gestational and childhood diabetes, management of diabetic complications, use and cost of medical treatment, blood glucose management, exercise treatment, COVID-19 vaccine and complications, and diabetes in older adults. The top three verb categories by noun topics were permission, method, and possibility. This study provided baseline data that can be used by clinical nurses to deliver diabetes-related education and management based on information sought by patients.

## 1. Introduction

Coronavirus disease 2019 (COVID-19) is a viral disease that was first identified on 1 December 2019 in Wuhan, a city in Hubei Province, China. Since then, there have been over 92 million confirmed cases and over 1.98 million deaths around the world [1]. The World Health Organization declared it a global pandemic [2], and despite the national emergency responses implemented by each country for infectious disease control, COVID-19 has continued to affect numerous people globally, and there is heightened concern about the emergence of new variants with greater infectivity around the world [3]. The clinical features of COVID-19 vary from the absence of symptoms to fever, coughing, breathing difficulty, and diarrhea. Although the initial symptoms may be mild, progression to a severe form of the disease could lead to death, particularly in patients with pre-existing conditions, such as diabetes [4].

During the COVID-19 pandemic, public health centers and public hospitals responsible for providing healthcare to patients with diabetes were designated as COVID-19 hub hospitals. Consequently, patients with diabetes faced difficulties with adherence to medication and participation in consultation and diabetes education sessions, due to the financial burden of having to use private hospitals [5]. Moreover, delayed diagnosis of diabetes caused a delay in active management and was conducive to the onset of complications. The diagnosis rate of type 1 diabetes mellitus (T1DM) decreased by 23% in 2020, relative to that in 2019, while 44.3% of patients with a first-time diagnosis of T1DM also had diabetic ketoacidosis [6]. In particular, 5.8% of patients with diabetes in Republic of Korea did not receive or delay their outpatient care during the COVID-19 pandemic [7]. Patients with diabetes represent a group vulnerable to infectious diseases, such as influenza and pneumonia, and when infected with severe acute respiratory syndrome coronavirus 2 (SARS-CoV-2), patients with diabetes have a 2.12-fold higher risk of COVID-19-related death and a 2.45-fold higher risk of progression to severe COVID-19 than healthy individuals [8]. Despite the need for strict healthcare and health information for self-care in daily life during the pandemic, the COVID-19-related measures restricted the necessary access [9].

Diabetes self-care refers to taking medications for the disease, controlling the diet, monitoring blood glucose, performing foot care and exercise, and refraining from smoking [10]. Diabetes self-care is related to many aspects of activities of daily living. Consequently, patients with diabetes seek information to resolve various questions they have about their own healthcare [11], as having access to additional disease-related information can increase treatment adherence and enhance self-care ability, which can be an important factor in preventing complications in patients with diabetes [12]. In addition, when patients with diabetes are unable to understand the information provided by healthcare professionals fully, they tend to seek additional information from other sources, such as family members and acquaintances, while they also spend much time in online searches, which are less time-consuming and have fewer spatial constraints [13].

Social lockdown in response to COVID-19 resulted in less time spent on walking and physical activities, while increasing sedentary time and the consumption of foods and snacks containing sugar [14]. Patients with diabetes have reported that they experienced a lack of physical activities and dietary management since the start of the COVID-19 pandemic [15]. While patients with diabetes developed elevated glycated hemoglobin (HbA1c), blood glucose, low-density lipoprotein cholesterol, and triglyceride levels during social lockdown, they also faced difficulties in receiving professional care for these elevated levels as well as in obtaining education or information for self-care in daily life [16]. The worsening of diabetes during this pandemic due to a decline in patients’ ability to self-manage their disease during the COVID-19-related social lockdown [15] highlights the need for providing more specific and diverse information to patients with diabetes during such an epidemic. In this respect, it is also necessary to identify what information patients with diabetes require to practice self-care. A review of studies on patients with diabetes during the COVID-19 pandemic showed the impact of social lockdown policies on glycemic control in patients with diabetes [17]; the immunity and response to COVID-19 of patients with diabetes [18]; COVID-19-related mortality rate among patients with diabetes [19]; and depression and fear related to COVID-19 [20]. In Republic of Korea, a study has reported on the unmet needs of patients with diabetes according to income [21], but studies related to health information for self-care among patients with diabetes in Republic of Korea are limited.

For healthcare professionals, identifying the information needs of patients and providing appropriate and accurate information to patients are important aspects of patient care [22]. Because interactions between patients and healthcare professionals during an epidemic are limited, it is necessary for healthcare professionals to provide reliable healthcare information to patients with diabetes to help them maintain self-care during daily life [17]. To this end, the health information that patients need for self-care during an epidemic must be identified. Analyzing questions asked by patients is one method that can enable the timely identification of the health information needs of these patients [23]. The bidirectional encoder representation from transformers (BERT) topic model is an analytical method with outstanding performance for natural language inference and analysis of Q&A records [24]. The BERT topic model can identify topics with latent meaning within text by means of text clustering [25]. It can extract topics more effectively than latent Dirichlet allocation [26], and thus, it is effective for analyzing the questions of patients with diabetes.

Accordingly, this study aimed to collect diabetes-related questions posted in an online community website during the COVID-19 pandemic to analyze the major keywords for diabetes-related health information, for the purpose of identifying the types of diabetes-related health information patients required and for providing basic data reflecting such health information.

## 2. Materials and Methods

### 2.1. Research Design

This text analysis study aimed to collect and analyze diabetes-related questions posted on an online diabetes-related community website during the COVID-19 pandemic to identify diabetes-related health information for the purpose of providing basic data to clinical nurses who care for patients with diabetes for devising self-care education plans.

### 2.2. Data Collection

The data used in this study consisted of diabetes self-care-related questions posted on a diabetes-related online community website. The online community website was selected based on the traffic volume, number of members, and number of posts. The online community website that was ultimately selected had 200,000 members and an average of approximately 120,000 posts per month. The data were used with permission from the administrator of the website. The data collection period was from 1 December 2019, which was the stage of concern regarding COVID-19 in Republic of Korea, to 3 May 2022, when the requirement for indoor mask wearing was officially lifted. The data were collected based on the title of the question, contents of the question, and posted date appearing in the bulletin board for “comprehensive counseling” within the diabetes consultation section of that website. Among a total of 9316 posts, a total of 9155 posts were used in the final analysis after excluding posts that were not related to diabetes.

### 2.3. Data Preprocessing

**Figure 1:**
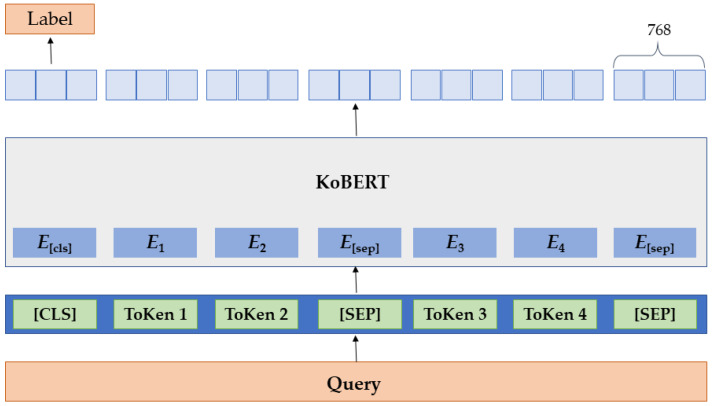
Architecture of KoBERT.

**Figure 2:**
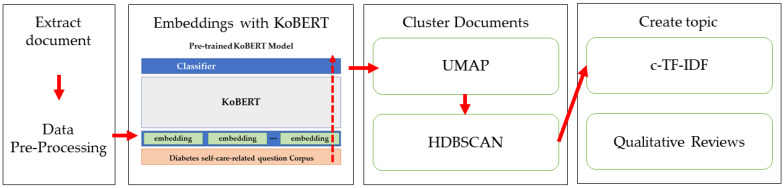
KoBERT topic modeling algorism.

The present study used BERT topic modeling and the data analysis procedures involved data preprocessing, document embedding, document clustering, and topic extraction. The specific details are described below. The natural language processing of the collected data consisted of pruning, tokenization, normalization, and stop-word removal, which refers to a series of processes, such as the removal of stop words, including special characters, symbols, and meaningless words in the posts, as well as the correction of spacing and grammatical revision. Subsequently, a unification of duplicate and blending words among words extracted using frequency analysis was required. In this study, PyKoSpacing, a package for automatic Korean word spacing, was used to correct spacing errors and the Python “replace” command was used to revise typos and misspelled English expressions and keywords, after which Python regular expressions were used to remove special characters, including emojis. Moreover, the Python module Mecab, a Korean morpheme analyzer, was used to add a user dictionary of compound words and words with a common meaning were substituted and unified based on the Python dictionary, followed by the removal of stop words to complete the data preprocessing. In this study, the Ko-sentence-transformers model was used for sentence embedding. The Ko-sentence-transformers model (Figure 1), a type of KoBERT for processing the Korean language, is a semisupervised learning model that can be applied to various natural language processing tasks. BERT limits the number of characters that can be included in one segment to a maximum of 512 characters. Table 1 shows a KoBERT-based solution. Thus, embedding was performed after dividing the length of the sentence into 512 characters to create a list. For document clustering, the uniform manifold approximation and projection (UMAP) algorithm was used to reduce the high dimensionality of local structure to a lower dimensionality after sentence embedding. N_neighbors is a hyperparameter that affects the performance of UMAP. Generally, a range of 5–50 is applied for n_neighbors, while a range of 10–15 is recommended as the most reasonable range [27]. In this study, the n_neighbors parameter was initially set to 10–15 in consideration of the data size, and after comparison of document clustering, n_neighbors was set to 15 and the metric was set to cosine. After structural dimensionality reduction using UMAP, a hierarchical density-based spatial clustering of applications with noise (HDBSCAN) was used for the final clustering. HDBSCAN includes the hyperparameter min_cluster_size [28], and in this study, a scatter plot of the clusters with min_cluster_size varying between 15 and 30 was checked, and the final analysis was performed by setting the min_cluster_size to 30. To check the specific details of the types of health information, noun and verb topics were extracted. English words and numbers were removed using Python regular expression, and noun topics were extracted via class-based term frequency–inverse document frequency (c-TF-IDF). c-TF-IDF is a method in which the sentences in each class are combined into a single document and the TF-IDF of the corresponding class is derived. This offers the advantage of demonstrating the topic of each class more accurately [29]. The overall analysis procedure is shown in Figure 2. In topic model analysis, qualitative analysis can be used to compensate for the limitations of quantitative analysis [30]. Thus, this study applied quantitative analysis using a scatter plot confirmed after HDBSCAN to select and interpret the appropriate number of topics, and a group of three experts (one nursing college professor, one doctor of nursing, and one nursing PhD student) was formed as a qualitative evaluation method to compensate for the limitations of quantitative analysis. For the interpretation of the topics derived through topic modeling, the experts repeatedly traced the data analysis results back to the original sentences and compared and contrasted them with the original data to exclude subjectivity in the meaning and interpretation of the topics as much as possible and to ensure the rigor of the topic names and interpretations. For the extraction of verb topics, high-frequency verbs were identified with c-TF-IDF. Among the extracted verbs, words with a common meaning were substituted and unified by the experts above, to check the categories of high-frequency verbs for each noun topic. To determine the categories of verbs, this study used the wh-questions theory proposed by Park [31].

### 2.4. Ethical Considerations

The present study received institutional review board review exemption (KHSIRB-22-455 [EA]).

## 3. Results

### 3.1. Noun Topic Modeling

**Figure 3:**
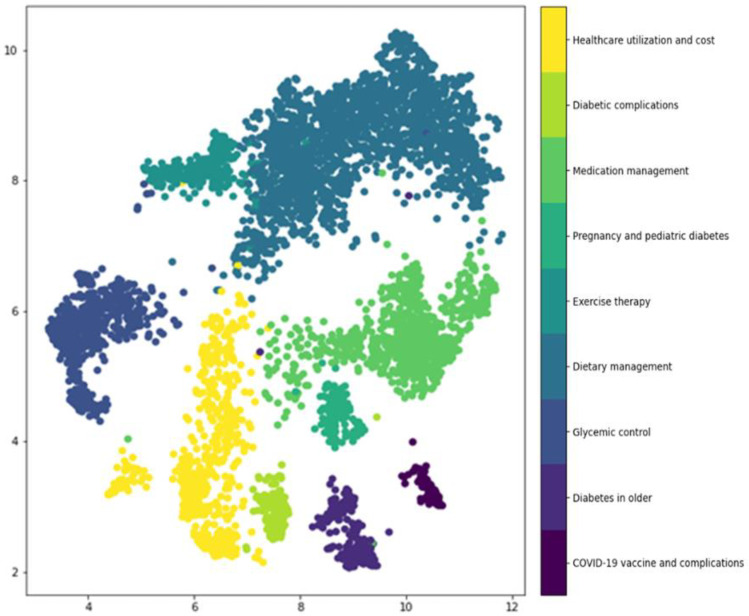
Scatter plot of document clustering.

When the UMAP algorithm and HDBSCAN were applied for document clustering, document clusters were formed as shown in Figure 3. Ultimately, nine clusters were selected in consideration of the meaning and ease of clustering. After checking c-TF-IDF of the derived clusters to identify the top 20 terms, the terms of the first topic were related to the COVID-19 vaccine and complications, including “COVID-19 vaccine”, “vaccination”, “thrombosis”, “antibody”, and “death”. The terms of the second topic were related to diabetes in older individuals, including “mother”, “father”, “insulin”, “management”, “hospital”, “surgery”, “age”, and “health”. The terms of the third topic were related to glycemic control, including “blood glucose monitor”, “use”, “blood glucose check”, “difference”, “lancet”, and “code”. The terms of the fourth topic were related to dietary management, including “after meal”, “diet”, “salad”, “carbohydrates”, “vegetables”, “eggs”, “side dishes”, “brown rice”, and “protein”. The terms of the fifth topic were related to exercise therapy, including “exercise”, “squat”, “knee”, “bicycle”, “aerobic exercise”, and “treadmill”. The terms of the sixth topic were related to pregnancy and pediatric diabetes, including “pregnancy”, “childbirth”, “children”, “babies”, “management”, “examination”, “HbA1c”, “lactation”, “breast milk”, and “impaired glucose tolerance”. The terms of the seventh topic were related to medication management, including “oral hypoglycemic agents”, “dosage”, “prescription”, and “nutritional supplements” and “side effects”. The terms of the eighth topic were related to the management of diabetic complications, including “pain”, “toenails”, “toes”, “neuropathy”, “sensation”, and “inflammation”. The terms of the ninth topic were related to healthcare utilization and cost, including “insurance”, “examination”, “hospital”, “ophthalmology”, “dispute”, “surgery”, “enrollment”, and “treatment” (Table 2). Based on this, the title of the topics for each cluster were assigned as COVID-19 vaccine and complications; diabetes in older individuals; glycemic control; dietary management; exercise therapy; pregnancy and pediatric diabetes; medication management; the management of diabetic complications; and healthcare utilization and cost. The frequency of document for each topic was as follows: dietary management, 2243 cases (26.5%); medication management, 1327 cases (15.7%); pregnancy and pediatric diabetes, 1140 cases (13.5%); management of diabetic complications, 996 cases (11.8%); healthcare utilization and cost, 925 cases (10.9%); glycemic control, 803 cases (9.5%); exercise therapy, 458 cases (5.4%); COVID-19 vaccine and complications, 303 cases (3.6%); and diabetes in older individuals, 270 cases (3.2%) (Table 3).

### 3.2. Verb Categories by Noun Topics

Major verb categories by noun topic are shown in Table 4. The frequency of questions for each topic appeared in the following order: permission (n = 99, 32.1%), method (n = 92, 29.9%), and possibility (n = 47, 15.3%) for the topic of COVID-19 vaccine and complications; method (n = 118, 43.7%), possibility (n = 39, 14.4%), and definition (n = 33, 12.2%) for the topic of diabetes in older individuals; method (n = 293, 36.5%), possibility (n = 127, 15.8%), and permission (n = 109, 13.6%) for the topic of glycemic control; permission (n = 906, 40.4%), method (n = 695, 29.4%), and possibility (n = 220, 9.8%) for the topic of dietary management; method (n = 222, 48.5%), permission (n = 87, 19.0%), and possibility (n = 68, 14.8%) for the topic of exercise therapy; method (n = 494, 43.3%), permission (n = 276, 24.2%), and possibility (n = 160, 14.0%) for the topic of pregnancy and pediatric diabetes; permission (n = 488, 36.8%), method (n = 459, 34.6%), and possibility (n = 155, 11.7%) for the topic of medication management; method (n = 396, 39.8%), permission (n = 264, 26.5%), and possibility (n = 132, 13.3) for the topic of management of diabetic complications; and method (n = 437, 47.2%), possibility (n = 157, 17.0%), and permission (n = 136, 14.7%) for the topic of healthcare utilization and cost.

## 4. Discussion

The present study aimed to discuss the findings derived through BERT topic modeling and cTF-IDF performed on diabetes-related questions posted in an online community website during the COVID-19 pandemic. BERT is bidirectional, unlike existing NLP models that were “unidirectional”, and uses MLM techniques to predict a specific word based on other words that exist in the sequence, so that the model learns the context which is said to understand. However, it can be difficult to understand the context, which is the main purpose of MLM, because randomly assigned masked words can select meaningless words rather than main words. In an effort to compensate for these shortcomings, this study applied a qualitative evaluation method using experts. Nine topics were finally extracted via noun topic modeling. The topics were similar to some items in the information provision categories of the Korean Diabetes Association (KDA), including “nutritional management”, “drug therapy”, and “diabetic complications”. Zhou and Ni [22] identified “diagnosis”, “treatment”, “lifestyle”, “complications”, “maternity”, “disease course”, “healthcare professionals”, “prevention”, and others via clustering diabetes-related questions posted on a Chinese health information website (http://www.39.net, accessed on 30 May 2022), among which the topic of “complications” was consistent with the topics in the present study. A study by Xusheng and Likeng [32] identified “body mass index”, “HbA1c”, “hypertension”, and “creatinine” by analyzing diabetes-related questions posted in Channel 39, a website operated by experts, which were different from the topics identified in the present study. Such differences could be attributed to the fact that the data in the present study were collected during the COVID-19 pandemic, and as a result, there may be some differences from data that were generated prior to the COVID-19 pandemic. Moreover, questions posted in the counseling section of the KDA website were rearranged and categorized by different fields, whereas the study by Xusheng and Likeng [32] collected questions from a diabetes-related expert website instead of a website for the general public. The present study confirmed that the classification of questions may vary depending on the questions frequently asked in specific situations and during particular periods, and that it may also vary according to the characteristics of the website users. Accordingly, nurses should also consider the environment and characteristics of patients with diabetes and changes in society when providing care to patients with diabetes as a clinical practice.

With respect to the specific details of each noun topic, the terms belonging to the topic of the COVID-19 vaccine and complications were “COVID-19 vaccine”, “vaccination”, “appointment”, “complications”, “thrombosis”, “pain”, and “medication”, indicating a high demand for information regarding COVID-19 vaccination and adverse events. While direct comparison is difficult due to a lack of existing studies on this topic, SARS-CoV-2 is known to increase the severity of COVID-19 in patients with chronic underlying diseases, and in particular, patients with diabetes face a higher risk of developing serious complications, being hospitalized, and progressing to severe COVID-19 than patients without diabetes. Additionally, a new onset of diabetes as a complication of COVID-19 has also been reported [8,9]. Therefore, patients with diabetes may have had high demand for information regarding COVID-19 prevention and infection control methods during the COVID-19 pandemic. The American Diabetes Association and the Johns Hopkins Patient Guide to Diabetes provide COVID-19-related information to patients with diabetes through their own websites [33,34] in efforts to reduce anxiety about emerging infectious disease among patients with diabetes [31]. However, patients with diabetes in Republic of Korea faced many limitations in obtaining relevant information from healthcare professionals during this pandemic [5], and as a result, they may have attempted to obtain COVID-19-related information through easily accessible online communities [35,36].

The terms included in the topic of diabetes in older individuals consisted of “mother”, “father”, “hypoglycemia”, “insulin”, “shock”, “complications”, and “treatment”, which indicated that guardians faced many difficulties in their care for a family member who uses insulin. Such findings were reconfirmed in studies by Dikkers and Dunning [37] and Holtz [38], which reported that the scope of providing diabetes-related health information needs to be expanded to include not only the patient but also the family members, due to the recent increase in the number of families caring for older patients with diabetes as well as the number of patients with diabetes. In particular, with the increased management of older patients with diabetes by family members during the social lockdown period caused by the COVID-19 pandemic, the demand for diabetes self-care information by means of websites also increased. In particular, in the unique situation created by an outbreak of an infectious disease, when access to healthcare professionals may not be easy, obtaining unreliable information through websites could cause other complications or progression to more severe disease. Considering these factors, it is necessary for certified institutions to provide information to patients with diabetes and their family to promote effective and efficient healthcare, while also providing information that family members can use for responding to any related emergency at home.

In the topic of glycemic control of patients with diabetes, the identified terms consisted of “blood glucose monitor”, “use of continuous glucose monitoring system”, “blood glucose check”, “finger”, “difference”, and “accuracy”, which indicated that patients with diabetes had many questions about the accuracy of and differences in blood glucose levels measured according to the type of blood glucose monitor and the site of blood glucose measurement. Previous studies have reported that blood glucose levels measured by a blood glucose monitor may vary depending on the type of blood glucose monitor used and the method and site of blood glucose measurement [39,40,41]. Such differences in blood glucose levels measured by means of a blood glucose monitor may be due to the performance of the device, but they can also occur due to personal factors (measurement method used and environmental factors) [39]. Patients can regularly visit hospitals for a correction of problems related to using a blood glucose monitor and for blood glucose measurements by healthcare professionals [42,43]. However, because of limitations in education through healthcare professionals during the COVID-19 pandemic, there was high demand for information related to blood glucose measurement.

The terms included in the topic of dietary management of diabetes consisted of “diet”, “salad”, “vegetables”, “eggs”, “side dishes”, “brown rice”, “protein”, “multigrain rice”, “tofu”, “breast meat”, “white rice”, and “soy milk”, with most of the questions being about specific types of food and consumption methods. In addition, the terms belonging to the topic of dietary management identified in the present study were after meal, fasting, before meal, blood glucose check, and highest blood glucose level, which indicated that patients with diabetes were paying close attention to changes in their blood glucose levels after meals. However, patients with diabetes faced difficulties due to a lack of knowledge about dietary management and planning, and, therefore, had a high demand for information and help from experts [44,45]. Patients with diabetes practice dietary control in daily life through relevant education received during hospital visits. However, diabetes education could not be conducted smoothly during the COVID-19 pandemic due to restrictions on hospital visits and because patients avoided hospital visits, which is likely why patients with diabetes attempted to acquire dietary management-related information through online communities. Moreover, changes in blood glucose levels of patients with diabetes are determined not only by the food consumed, but also by various factors, including climate change, insulin secretion, insulin sensitivity, the secretion of gastrointestinal hormones in response to ingested nutrients, stress, and mood changes [46,47]. Because of these reasons and due to inconsistent blood glucose measurements according to the type of food consumed, patients with diabetes experience difficulties in managing their diet [48,49]. Therefore, nurses and other healthcare professionals who provide education to patients with diabetes in clinical practice need to prepare and provide specific information regarding changes in blood glucose levels according to the type of food consumed by patients with diabetes, as well as about factors other than food that may affect changes in blood glucose levels.

For the topic of exercise therapy, the terms identified consisted of “exercise”, “squat”, “bicycle”, “indoor”, “aerobic exercise”, “stepper”, and “treadmill”, indicating a high demand for information regarding indoor exercise. Such findings were different from a study by Ramsingh and Bhuvaneswari [50] that analyzed website posts of patients with diabetes in India, which reported that “yoga” and “cycling” were the major terms related to exercise among patients with diabetes. It is believed that, because the data collection period in the present study was during a period when outdoor activities were restricted by social lockdown and distancing in response to COVID-19 [14], patients with diabetes may have shown a high demand for information regarding indoor exercise to control their blood glucose levels. In particular, in Republic of Korea, individuals experienced a lack of physical activities during the COVID-19 pandemic [15], which could have caused anxiety with respect to self-care among patients with diabetes. Therefore, particularly after the experience with this major pandemic, healthcare professionals should consider the need for providing information regarding indoor exercise to patients with diabetes. In particular, they should provide information about various indoor exercise programs that can be safely applied at home and should introduce such programs through the websites of applicable medical institutions.

The terms included in the topic of pregnancy and pediatric diabetes were mostly “pregnancy”, “childbirth”, “baby”, “breast milk”, and “lactation”. Such findings were slightly different from the keywords “exposure”, “fetus”, “hypoglycemia”, “marker”, medication”, “metabolic disease”, “preeclampsia”, “prevention”, “program”, and “smoking” identified in a text analysis study on gestational diabetes [50]. Unlike the present study, which analyzed texts regarding pregnancy and childbirth posted by individuals who are not healthcare professionals, based on a collection of questions posted in a diabetes-related online community website, the study by Lee and Kim [51] used data from previous studies on gestational diabetes, and as a result, many of the keywords were medical terms. Such findings confirmed that there are differences in gestational-diabetes-related information between patients and experts and that patients with diabetes have a broad range of information needs, including parenting-related information, such as breastfeeding while taking diabetes medication after giving birth. In particular, “children”, “diet”, “impaired glucose tolerance”, “hospital”, and “blood glucose check” appeared as the top terms of this topic, which reflected the fact that guardians who have children with diabetes focused on accepting and managing diabetes through understanding of the effects of diabetes on children and family, as well as the pathogenesis of diabetes. Therefore, sufficient information on this topic should also be provided.

For the diabetes medication management topic, the identified terms reflected the demand for information regarding medication taken by patients with diabetes. Metformin, which was one of the terms identified in the present study, is an oral drug for diabetes. This may have caused an increase in the frequency of questions regarding medication on diabetes-related health information websites. Therefore, health information for patients with diabetes should include information regarding drugs that have recently become social issues and health expert counseling methods related to drugs [52,53]. In particular, the present study focused on the period during the COVID-19 pandemic, and thus, opportunities for face-to-face encounters between patients and healthcare professionals were limited5. Consequently, questions regarding medication on websites operated by non-professionals, accounting for a significant portion of the questions regarding symptom management, were about drug misuse and abuse. Therefore, it is necessary to promote Q&A actively to enable patients to receive accurate information through websites operated by healthcare professionals or medical institutions. Allocating dedicated personnel for such tasks is also necessary.

The terms included in the topic of diabetic complications consisted of “pain”, “toenails”, “toes”, “soles”, “ankles”, “legs”, “big toe”, “orthopedics”, “callosity”, and “wounds”, indicating a high demand for information regarding diabetic complications, particularly diabetes-related foot diseases, among patients with diabetes. In a previous study [3], among diabetic complications, patients with diabetes showed the highest level of interest in ocular complications. This difference may be due to the difference in the methods by which the questions were analyzed. Wang and Zhou [3] extracted the frequency of terms that appeared most often for each topic in health-related websites, whereas the present study used the Ko-sentence-transformers model to extract keywords based on context. Therefore, the study by Wang et al. may have classified the topic terms by focusing simply on the terms, rather than considering the context of these terms, which may have resulted in keywords for patients with diabetes being reflected less commonly in their findings. Therefore, future studies analyzing diabetes-related questions should use various data sources and multiple methodologies for clustering collected questions and then compare the results. Based on our study and that of Wang and Zhou [3], there is a high demand for information regarding diabetic foot diseases and ocular complications among patients with diabetes

In the topic of healthcare utilization and cost, the terms identified consisted of “hospital”, “ophthalmology”, “retina”, “surgery”, “complications”, “diagnosis”, “treatment”, “appointment”, and “doctor”, and thus, it can be surmised that patients with diabetes requested information regarding hospitals and clinics. In particular, information regarding hospitals for ophthalmic care was identified as important, considering the contextual dimension. While direct comparison is difficult due to a lack of existing studies on this topic, it is suspected that patients with diabetes sought information about hospitals and clinics through websites because of restrictions on visits to these institutions due to social distancing implemented during the COVID-19 pandemic [54]. Moreover, considering that other keywords, such as “insurance”, “examination”, “actual expenses”, and “enrollment” were included in the topic of healthcare utilization and cost, it was determined that patients with diabetes have a high demand for information regarding healthcare costs. Patients who have been diagnosed with diabetes have increased healthcare utilization due to regular visits to hospitals/clinics, frequent examinations, and drug purchases [55]. As a result, they may face financial hardship [39] and patients with diabetes with diminished socioeconomic activities due to the COVID-19 pandemic may have shown even higher demand than normal for information regarding costs and support for their own healthcare.

For the noun topics identified in the present study, the questions were classified into categories of pediatric diabetes, gestational diabetes, and diabetes in older individuals, depending on the stage of life to which the question pertained. Such findings were consistent with a study on a needs survey for providing health information conducted on an Internet panel group, which reported that different population groups of respondents showed different preferences for health information [56]. This may be due to the characteristics of people who use online health information. According to a previous study, people who mostly use online health information are people who play the role of caregiver at home, such as married women [57,58]. In particular, patients with pediatric diabetes, gestational diabetes, or diabetes in older individuals represent a group of patients who require assistance and care from guardians, and thus, most of the questions on the website were posted by guardians. Thus, information provided by websites should include contents that can be helpful for not only patients but also guardians, as mentioned in the studies by Dikkers and Dunning [37] and Holtz [38]. Moreover, it is also necessary to plan more systematic education and operate programs in clinical practice not only for the patients with diabetes but also for their guardians. In addition, it is also necessary to prepare plans for providing health information to patients and guardians who may have a poor ability to use online information.

The findings in the present study showed that the top-4 verb categories of the noun topics were “permission”, “method”, “possibility”, and “definition”. Such findings were similar to “definition”, “method”, and “effect” that were identified as verb categories in previous studies that analyzed the types of product-related questions from purchasers of general electronic products [59,60]. The health information sought by patients with diabetes is thought to be reflected by questions seeking expert opinions on glycemic control, medication, and dietary management for self-care. General electronic products are related to diabetes in that purchasers would ask many questions about how to use the product on their own, as a non-expert. However, a prominent characteristic of health-related questions is the desire to acquire information that they currently lack, in order to increase their body of knowledge and to reduce the information gap with others [61]. Although healthcare professionals are making various efforts to provide high-quality health information to patients, patients still feel a deficiency in the information they need during their hospital/clinic visits or treatment process [62]. In particular, the verb category of “method” was extracted the most among the noun topics related to diabetes in older individuals, glycemic control, exercise therapy, pregnancy and pediatric diabetes, the management of diabetic complications, and healthcare utilization and cost. While a direct comparison is difficult due to the limited number of previous studies on the types of diabetes-related questions posted on online community websites, the findings in the present study were similar to the findings in a study by Chiu and Wu [63] on the analysis of 440 cases of health-related questions in Yahoo! Answers, which reported that questions related to specific methods (n = 235, 52%) accounted for the greatest proportion of questions. Among questions regarding health information, the type of questions requiring professional knowledge to which users cannot find the answers by simple searching accounted for a high percentage of questions [60]. Patients with diabetes face difficulties in coping with various situations in daily life in the absence of professional help, but they have a relative lack of specialized knowledge and information. Consequently, they have an increased tendency to make decisions based on the experiences of others. Therefore, health information for patients with diabetes should be more specific and practical to allow them to maintain management of their own health, even during various unexpected situations, such as an epidemic, when it is difficult to visit medical institutions or to perform in-person examination by healthcare professionals. In addition, there is a need to establish a system to provide easy access to such information, while the scope and amount of information provided by medical institutions, societies, and associations also need to improve.

In addition, the verb category for confirming “permission” was mostly found in the topics of COVID-19 vaccine and complications, dietary management, and medication management. This type of question seeks to obtain permission as an assurance or consent for their own actions under situations that require medical judgement. Exchange of such information by non-healthcare professionals could lead to drug misuse and abuse [53]. Online information users with a strong tendency to resolve health problems on their own have a high risk of self-assurance, such as making a diagnosis and seeking unnecessary prescription on their own, based on online health information searches, as well as not adhering to prescribed medication, with no communication with healthcare professionals [60]. In particular, during situations in which resolving problems through consultation with a healthcare professional is difficult due to the COVID-19 pandemic [64], the tendency for self-determination regarding health problems could be unintentionally further reinforced. However, online health information is generated and distributed by both healthcare professionals and non-healthcare professionals, and thus, a problem with the reliability of information exists [65,66]. Accordingly, it is necessary to strike a balance between self-determination of health information users, further reinforced by COVID-19, and disease management through healthcare professionals. An educational system could also be contemplated based on the present study. In particular, healthcare professionals need to shift from the conventional paradigm of thinking of themselves as a provider of treatment and information to patients with diabetes to a concept of being partners in the patients’ disease process [67]. Moreover, healthcare professionals need to pay attention to information generated by patients with diabetes through community websites and should provide sufficient information to enable patients with diabetes to make their own decision regarding problems that do not require medical judgement. They also need to design an effective information architecture to support user access to various types of information. Furthermore, a decision-making protocol that can help identify problems that require medical judgement, among various problems faced by patients with diabetes during the course of their disease, is also needed. Through such methods, the ability to cope with diabetes and the self-efficacy of health information users could be enhanced [68]. The unmet health information need of patients with diabetes can be satisfied by providing information and stable counseling via healthcare professionals.

## 5. Limitation

The present study analyzed posts from a single website, and thus, the study was limited in identifying questions according to the characteristics of websites or medical institutions. Therefore, future studies using a text analysis of diabetes-related questions extracted from posts in various types of websites are needed. In the case of topic analysis, since the analysis data and algorithms used by each researcher are diverse, we suggest a future study to consistently compare the meaningful results between the results of this study and previous studies.

## 6. Conclusions

The present study collected and analyzed diabetes-related questions posted on a website to identify the health information needs of patients with diabetes during the COVID-19 pandemic for the purpose of providing basic data for self-care by patients with diabetes. The present study was significant in that it analyzed diabetes-related health information requested by online community users to derive the topics for diabetes-related health information and provided basic data regarding health information needs of patients with diabetes. The present study was also significant in that it used topics related to diabetes-related health information derived in the study to provide basic data for developing self-care education programs for patients with diabetes. However, in order for the model developed in this study to have more sophistication and generalization, it should be fine-tuned and tested. In future studies, it is also expected to develop diabetes-related educational contents based on the results of this study.

## Figures and Tables

**Table 1:** KoBERT-based Solution.

Attribute	Value
Features	768
Hidden size	3072
Number of layers	12
Max_length	512
Number of classes	2
Dropout	0.1

**Table 2:** Top 20 terms by noun topic.

Ranking	COVID-19 Vaccine and Complications	TF-IDF	Diabetes in Older Individuals	TF-IDF	Glycemic Control	TF-IDF	Dietary Management	TF-IDF	Exercise Therapy	TF-IDF	Pregnancy and Pediatric Diabetes	TF-IDF	Medication Management	TF-IDF	Management of Diabetic Complications	TF-IDF	Healthcare Utilization and Cost	TF-IDF
1	COVID-19 vaccine	0.58	mother	0.09	blood glucose monitor	0.17	after meal	0.04	exercise	0.16	pregnancy	0.14	oral hypoglycemic agents	0.09	pain	0.09	insurance	0.05
2	vaccination	0.20	father	0.07	use	0.07	diet	0.03	squat	0.08	childbirth	0.09	dosage	0.06	toenails	0.09	examination	0.05
3	reservation	0.11	hypoglycemia	0.03	blood glucose test strips	0.06	fasting	0.03	knee	0.07	children	0.07	prescription	0.04	toes	0.08	hospital	0.05
4	injection	0.05	insulin	0.03	buy	0.06	snack	0.02	bicycle	0.05	babies	0.04	side effects	0.03	neuropathy	0.07	ophthalmology	0.03
5	patient	0.05	management	0.02	continuous glucose meter	0.05	salad	0.02	muscular strength	0.05	management	0.03	HbA1c	0.03	sole of the foot	0.06	retina	0.03
6	prevention	0.04	hospital	0.02	purchase	0.04	blood glucose check	0.02	fitness center	0.05	examination	0.03	nutritional supplements	0.03	sensation	0.06	clinic	0.03
7	side effects	0.04	diet	0.02	consume	0.04	before meal	0.02	inside	0.04	normal	0.03	blood pressure	0.02	leg	0.05	actual expense	0.03
8	flu	0.04	prescription	0.02	blood glucose check	0.04	carbohydrates	0.02	after meal	0.04	fasting	0.03	hyperlipidemia	0.02	peripheral	0.05	surgery	0.03
9	thrombosis	0.03	doctor	0.02	difference	0.04	high blood sugar	0.02	aerobic exercise	0.04	HbA1c	0.03	vitamin	0.02	injury	0.05	complications	0.03
10	hypertension	0.03	surgery	0.02	prescription	0.04	vegetables	0.02	effect	0.03	insulin	0.02	doctor	0.02	treatment	0.04	enrollment	0.03
11	hyperlipidemia	0.02	take	0.02	material	0.03	eggs	0.02	stairs	0.03	lactation	0.02	stopping the pill	0.02	inflammation	0.03	HbA1c	0.02
12	pain	0.02	blood pressure	0.02	recommendation	0.03	side dishes	0.02	stepper	0.03	diet	0.02	effect	0.02	part	0.03	treatment	0.02
13	children	0.02	shock	0.02	pharmacy	0.03	brown rice	0.01	treadmill	0.03	breast milk	0.02	after meal	0.02	dermatology	0.03	reservation	0.02
14	take	0.02	complications	0.01	lancet	0.03	protein	0.01	diet	0.03	impaired glucose tolerance	0.02	diet	0.02	circulation	0.03	actual expense	0.02
15	antibody	0.02	treatment	0.01	finger	0.03	multigrain rice	0.01	thigh	0.03	hospital	0.02	management	0.02	complications	0.03	doctor	0.02
16	death	0.02	diagnosis	0.01	code	0.02	tofu	0.01	blood glucose check	0.02	blood glucose check	0.02	fasting	0.01	ankle	0.03	diagnosis	0.02
17	disease	0.02	fasting	0.01	price	0.02	chicken breast	0.01	deadweight	0.02	after meal	0.02	weight	0.01	hands and feet	0.03	general	0.02
18	news	0.02	health	0.01	accuracy	0.02	polished rice	0.01	posture	0.02	ob-gyn	0.02	hypoglycemia	0.01	big toe	0.03	laser	0.01
19	muscle pain	0.01	age	0.01	finger prick	0.02	soybean milk	0.01	waist	0.02	exercise	0.01	before meal	0.01	orthopedics	0.03	special	0.01
20	danger	0.01	weight	0.01	support	0.02	bowl	0.01	too much	0.02	diabetes	0.01	lactobacillus	0.01	corn	0.02	nature of a disease	0.01

TF-IDF = frequency–inverse document frequency.

**Table 3:** Examples of questions and frequency of noun topics.

Topics	Examples of Questions	n (%)	
COVID-19 vaccine and complications	Can I get vaccinated? After getting the COVID-19 vaccine, can I still take insulin as usual? I have been diagnosed with COVID-19. Do I need to take medication? Do blood glucose levels change after vaccination?	308	(3.6)
Diabetes in older individuals	My mother has diabetes. Is herbal medicine prohibited? My mother suffered hypoglycemic shock. Please advise. Can you take a look at the fasting blood glucose level of my mother who is in her 80s? Please recommend nutritional supplements for my mother who is in her 60s.	270	(3.2)
Glycemic control	What is a good blood glucose monitor? Can I get diabetes consumables even on Saturdays? Is there a relationship between blood glucose monitoring and temperature? Please tell me where I can buy test papers near Sokcho City Hall.	803	(9.5)
Dietary management	Can I eat rice (brown rice, multigrain rice)? Can I continue to drink bitter melon and pig potato decoction? What type of noodles should I eat? Is there any delivery food that I can eat?	2243	(26.5)
Exercise therapy	How should I manage my blood glucose level without losing weight? When my fasting blood glucose level is high, can I exercise on an empty stomach? Is ankle pumping exercise bad for peripheral neuropathy? Squats and walking after dinner should be the main exercise, right?	458	(5.4)
Pregnancy and pediatric diabetes	Is there anyone who had diabetes before pregnancy and stopped breastfeeding after childbirth? How did you stop breastfeeding? How are you controlling the blood glucose levels of your child? If postprandial blood glucose levels are high at week 16 of pregnancy, should I get tested for gestational diabetes right away?	1140	(13.5)
Medication management	Can I take anti-inflammatory painkillers if I have a headache? Can I take diabetes medicine and nutritional supplement together? Which drug should I take if I want to reduce side effects? Is constipation a side effect of Diabex XR extended release tablet?	1327	(15.7)
Management of diabetic complications	Can a toe bruise lead to necrosis? Can overwork cause diabetic complications? Are myalgia, fever, and diarrhea symptoms of acute diabetes? Could deterioration of peripheral nerves be due to blood glucose level?	996	(11.8)
Healthcare utilization and cost	Is there a hospital in Bundang that is recommended for kidney tests? Are there any dental clinics good at treating patients with diabetes? Is there any private health insurance that patients with diabetes can enroll in? Which department (hospital) should I go to for impaired fasting glucose test?	925	(10.9)

**Table 4:** Examples of questions related to major verb categories by noun topic.

Topics	Major Verb Categories	Examples of Questions
COVID-19 vaccine and complications	(Decision questions) permission	Can I take diabetes medicine after being diagnosed with COVID-19?
	(Question word questions) method	What should I do about my blood glucose level that went up after being diagnosed with COVID-19?
	(Decision questions) possibility	Is it possible for my blood pressure to go up after vaccination?
Diabetes in older individuals	(Question word questions) method	How should I control my mother’s blood glucose level?
	(Decision questions) possibility	Is it possible that my father can have an elevated glycated hemoglobin level because of gaining weight?
	(Question word questions) definition	My mother has hypertension. What are the symptoms of hypertension?
Glycemic control	(Question word questions) method	How should I dispose of a lancet?
	(Decision questions) possibility	If I am using a Libre, is there bleeding when exchanging the Libre?
	(Decision questions) permission	Can I use it past the expiration date?
Dietary management	(Decision questions) permission	Can I drink squeezed juice?
	(Question word questions) method	What proportion of rice should I eat?
	(Decision questions) possibility	Can blood glucose levels go up after eating sugar-free snacks?
Exercise therapy	(Question word questions) method	My blood glucose level keeps going up. What exercise should I do?
	(Decision questions) permission	Can I do indoor cycling exercise? There are many who say walking exercise is the best.
	(Decision questions) possibility	After anaerobic (muscle strengthening) exercise, can I experience sharp pain in that area?
Pregnancy and pediatric diabetes	(Question word questions) method	My child has high blood glucose levels. How should I manage the blood glucose levels? How should I prepare for pregnancy after being diagnosed with diabetes?
	(Decision questions) permission	Can I breastfeed after giving birth even if I have diabetes?
	(Decision questions) possibility	I am pregnant. Is there a possibility that diabetes can be passed down to my child?
Medication management	(Decision questions) permission	Can I take complete nutritional supplements?
	(Question-word questions) method	How should I take metformin and statins?
	(Decision questions) possibility	Does Champix raise blood glucose levels? I am taking Champix to quit smoking, but my blood glucose levels went up a lot.
Management of diabetic complications	(Question word questions) method	What are some methods for coping with hypoglycemic shock?
	(Decision questions) permission	During a neurological examination due to foot numbness, can I tell them that I have diabetes before the examination?
	(Decision questions) possibility	If my blood glucose level is managed well, will I see improvement in cold hands and feet?
Healthcare utilization and cost	(Question word questions) method	How does 00 hospital manage patients with diabetes?
	(Decision questions) possibility	Can medication expenses be claimed from private insurance?
	(Decision questions) permission	Can I be treated by an endocrinologist?

## Data Availability

The datasets used and/or analyzed during the current study are available from the corresponding author on reasonable request.
